# Contrast-enhanced vs. standard endoscopic ultrasound fine-needle aspiration for diagnosing malignant biliary tumors: Randomized controlled trial

**DOI:** 10.1055/a-2569-8969

**Published:** 2025-05-12

**Authors:** Rares Ilie Orzan, Sorana D. Bolboacă, Cristina Pojoga, Claudia Hagiu, Ofelia Mosteanu, Ioana Rusu, Voicu Rednic, Radu Seicean, Nadim Al Hajjar, Renata Agoston, Andrada Seicean

**Affiliations:** 1 433709 Gastroenterology, Regional Institute of Gastroenterology and Hepatology Prof Dr Octavian Fodor, Cluj-Napoca, Romania; 2 37576 Medical Informatics and Biostatistics, Iuliu Hațieganu University of Medicine and Pharmacy, Cluj-Napoca, Romania; 3 Clinical Psychology and Psychotherapy, Babeș-Bolyai University, Cluj-Napoca, Romania; 4 37576 3rd Department of Internal Medicine, Iuliu Hațieganu University of Medicine and Pharmacy, Cluj-Napoca, Romania; 5 3rd Medical Clinic, University of Medicine and Pharmacy Cluj-Napoca, Cluj-Napoca, Romania; 6 433709 Regional Institute of Gastroenterology and Hepatology Prof Dr Octavian Fodor, Cluj-Napoca, Romania; 7 37576 Iuliu Hatieganu University of Medicine and Pharmacy, Cluj-Napoca, Romania; 8 Regional Institute of Gastroenterology and Hepatology, University of Medicine and Pharmacy, Cluj-Napoca, Romania

**Keywords:** Endoscopic ultrasonography, Biliary tract, Fine-needle aspiration/biopsy, Tissue diagnosis

## Abstract

**Background and study aims:**

Contrast-enhanced endoscopic ultrasound (CH-EUS) is superior to standard EUS for staging biliary duct tumors (BDTs), but its role in guiding EUS-guided fine needle aspiration (EUS-FNA) remains unclear. We compared diagnostic accuracy of CH-EUS-fine needle aspiration (CH-EUS-FNA) and standard EUS-FNA in patients with suspected malignant biliary stenosis.

**Patients and methods:**

A parallel randomized controlled trial was conducted in a tertiary medical center and included jaundiced patients with suspected malignant biliary stenosis on computed tomography. The patients were assigned randomly to EUS-FNA or CH-EUS-FNA groups. Final diagnosis was determined based on EUS-FNA, surgical specimen results, endoscopic retrograde cholangiopancreatography (ERCP), or 12-month follow-up.

**Results:**

Sixty-one patients were included in the study, 31 in the EUS-FNA group and 30 in the CH-EUS-FNA group. Mean age of participants was 74 ± 11.04 years and mean tumor size was 20.39 ± 9.17 mm, with 43 tumors in the distal bile duct. Final diagnoses were cholangiocarcinoma (37 cases), pancreatic ductal carcinoma (12 cases), other malignancies (3 cases), and benign lesion (9 cases). Diagnostic sensitivity, specificity, and accuracy were 83.3%, 100%, and 87.1% for EUS-FNA, and 82.1%, 100%, and 83.3% for CH-EUS-FNA. Plastic biliary stent placement and tumor location did not influence results. Hyperenhancement in the CH-EUS with rapid washout was observed in 90.9% of cholangiocarcinoma cases.

**Conclusions:**

Standard EUS-FNA and CH-EUS-FNA demonstrated comparable diagnostic accuracy in evaluation of extrahepatic bile duct tumors, but with better slightly efficiency and inaccuracy indices than standard EUS-FNA.

## Introduction

Cholangiocarcinoma (CCA) is a rare malignant tumor that accounts for 10% to 20% of hepatobiliary malignancies and is one of the leading causes of extrahepatic biliary stenosis 1 . Due to its aggressive nature and limited treatment options 2 , the 5-year survival rate is only 10% to 40%, largely because tumors are often diagnosed at an advanced stage, reducing the possibility of surgical resection 3 . Distinguishing CCA from other malignant stenoses, such as bile duct lymphoma, gallbladder carcinoma, metastatic disease, pancreatic cancer, or ampullary tumors, is crucial. Benign diseases that can mimic malignant proximal strictures include IgG4 cholangiopathy, primary sclerosing cholangitis, eosinophilic cholangitis, biliary papillomatosis, infection response, trauma, ischemia, and chronic pancreatitis 4 .

Endoscopic ultrasound (EUS) can detect and characterize biliary extrahepatic tumors as either a mass or an extrahepatic stricture 5 . The sensitivity (Se) of EUS for detecting distal CCAs ranges from 79% to 89%, and from 57% to 68% for perihilar CCAs 6 7 .In adition, EUS allows for tumor sampling to provide a cytological diagnosis 8 with sensitivity (Se), specificity (Sp), and diagnostic accuracy (Acc) of 73.9%, 100%, and 80%, respectively 9 .

Usefulness of contrast enhancement in such lesions is less studied. Meacock et al. showed CCAs as hyperenhanced lesions with rapid washout on contrast-enhanced EUS (CH-EUS) 10 . Otsuka et al. assessed tumor staging and reported better results compared with conventional EUS, especially in terms of invasion beyond the biliary wall into surrounding tissues 11 . Similar to transabdominal ultrasound, CH-EUS enables precise guidance for fine needle aspiration (FNA) procedures, excluding necrotic areas and vascular elements within lesions 12 . The role of CH-EUS in guiding tissue acquisition in CCAs is not well understood, although previous studies in pancreatic solid lesions showed no significant difference when EUS-FNA/biopsy was performed with or without contrast guidance 13 .

The primary outcome of our prospective randomized study was to evaluate diagnostic accuracy of CH-EUS-FNA compared with standard EUS-FNA in diagnosis of extrahepatic bile duct tumors. Secondary outcomes included the impact of tumor location, enhancement patterns, and presence of stents on diagnostic performance.

## Patients and methods

### Patients

This randomized controlled trial was conducted at the Regional Institute of Gastroenterology and Hepatology Cluj-Napoca. The study protocol adhered to the 1975 Declaration of Helsinki guidelines, it was approved by the Ethics Committee of the hospital, and it was registered on ClinicalTrials.gov. Consecutive patients with suspected malignant extrahepatic bile duct neoplasms, recruited from November 2021 to February 2023, were randomly assigned to one of two parallel groups: EUS-FNA or CH-EUS-FNA. Inclusion criteria were: 1) presence of a bile duct tumor identified on imaging (computed tomography [CT], magnetic resonance imaging [MRI], or needing EUS-FNA); 2) age 18 years or older; and 3) ability to provide written consent. Exclusion criteria were: 1) bleeding tendency (international normalized ratio [INR] > 1.5 and/or platelet count < 50,000/mm³); 2) history of previous treatment for hepato-biliary tumors; 3) previous biliary surgery except bilioenteric drainage; 4) patient refusal to participate; 5) duodenal stenosis impeding full examination of the common bile duct; 6) operable proximal biliary tumors; and 7) presence of metallic biliary stents. Patients with incomplete follow-up after diagnosis were also excluded. In the case of a plastic stent placed before EUS-FNA, the stent was removed if lesion visibility on EUS was impeded and reinserted subsequently.

### Random allocation to procedure

Patients were enrolled as inpatients based on endoscopist assessment. A computer-generated randomization sequence, devised by a blinded statistician, was employed using a 1:1 schema ratio. Allocation to the study arms was concealed using sequential numbers. The number assigned to each patient determined their inclusion in either the EUS-FNA group or the CH-EUS-FNA group. Participants in the study and the pathologists were blinded to the assigned interventions during the study.

### Procedure algorithm

Following EUS assessment of solid lesions, two passes of EUS-FNA or CH-EUS-FNA were performed. Adequacy of samples was assessed using the Macroscopic On-Site Evaluation (MOSE) technique, considering puncture failure if core length was less than 5 mm. In such cases, a third pass of EUS-FNA was performed, but its result was not included in statistical analysis 14 . Final diagnosis was based on results of the initial EUS-FNA (two or three passes as necessary) or CH-EUS-FNA, a second procedure of EUS-FNA, endoscopic retrograde cholangiopancreatography (ERCP), postsurgical histopathological examination, or 12-month follow-up. Patients with negative FNA findings for malignancy were monitored for 12 months through clinical examinations and CT scans at 3 months, with subsequent follow-up as required.

### Procedure

For EUS examinations, we used a linear echoendoscope (Olympus GF-UCT 180 AL5; Olympus, Tokyo, Japan) combined with an ultrasound platform (Hitachi ARIETTA 850) and 22-G needles (Expect; Boston Scientific, Marlborough, Massachusetts, United States). All interventions were performed by experienced gastroenterologists (AS, CP, CH, OM) with over 1000 EUS-FNA and more than 200 CH-EUS procedures each. Patients were sedated with either light sedation (intravenous midazolam) or propofol sedation.

### Puncture

The puncture technique employed for the procedure involved utilizing both the fanning technique and 20-mL syringe suction, with two passes, using MOSE for sample evaluation. For CH-EUS-FNA patients, SonoVue (Bracco, Milan, Italy) was used as the ultrasound contrast agent, administered via an intravenous bolus injection of 2.4 mL followed by a 5-mL saline flush, as recommended by the European Federation of Societies for Ultrasound in Medicine and Biology 12 . A 7.5-MHz transducer with a mechanical index (MI) of 0.20 was utilized for the contrast procedure.

**Fig. 1:**
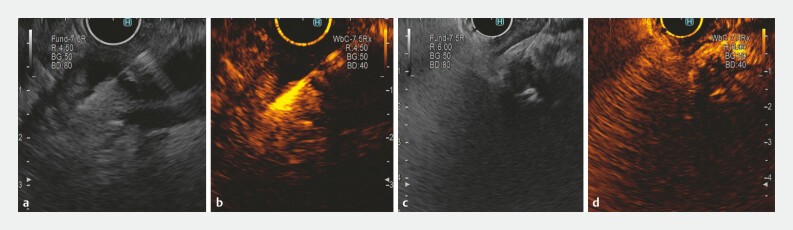
**a** EUS-FNA of a distal cholangiocarcinoma. **b** CH-EUS-FNA of the same distal lesion. **c** EUS-FNA view of a proximal hypoechoic cholangiocarcinoma. **d** CH-EUS-FNA of the same proximal lesion.

Following contrast injection, tumor enhancement compared with the surrounding tissue (hypoenhancement, isoenhancement, hyperenhancement) was evaluated during the arterial phase (25–30 seconds after SonoVue injection) and venous phase (30 seconds after injection). We also assessed the pattern of contrast uptake (homogeneous or heterogeneous) and rate of contrast washout (rapid or slow) ( Fig. 1 ). CH-EUS-FNA was performed in the same manner as EUS-FNA. At 45 seconds after contrast injection, the needle was inserted into the enhanced area of the targeted lesion, avoiding non-enhanced areas indicative of necrosis and large vessels within the lesion. Records of the echoendoscopic evaluation and biopsy acquisition were stored. The T stage was evaluated in the late phase following injection of SonoVue.

### Preparation of samples

No rapid on-site histological evaluation was performed. To preserve the core sample, the needle stylet was reintroduced, then flushed with saline, and the obtained tissue was expelled into a container with a 10% buffered formalin solution. This step ensured proper fixation and preservation of the core sample for subsequent cytological analysis. Samples obtained from each of the two passes were placed in the same container and analyzed together by the pathologist.

Following collection, specimens were embedded in paraffin, sectioned, and stained using the hematoxylin-eosin-safran (H&E) staining technique. In addition to H&E staining, some specimens underwent immunohistochemical staining. By employing both H&E staining and immunohistochemistry, a comprehensive evaluation of specimens was achieved, enabling a detailed examination of their cellular morphology, architecture, and presence of specific biomarkers.

Two blinded pathologists evaluated the FNA specimens. Histologic evaluations followed the Papanicolaou classification according to the new proposed terminology for pancreaticobiliary cytology, categorizing samples as nondiagnostic, negative for malignancy, atypia, neoplastic, suspicious, or malignant 15 . Diagnostic criteria for malignancy were as follows: 1) malignant cells or suspicion of malignant cells obtained by EUS-FNA or CH-EUS-FNA; 2) detection of malignancy through surgical specimens; 3) clinical symptoms or imaging modalities indicating progression during the follow-up period (at least 12 months); and 4) death due to malignancy. In the absence of malignant criteria, benign strictures were considered.

### Definitions

Proximal tumors were considered to be those located above the confluence with the cystic duct, whereas distal tumors were those located below the confluence but above the ampulla of Vater 16 . EUS features indicative of a mass included a lesion extending beyond the bile duct wall or exhibiting periductal infiltration, bile duct wall thickness exceeding 3 mm, or intraductal mass-forming lesion 5 . EUS criteria indicative of a malignant stricture were disruption of the trilaminar structure of the bile duct wall, presence of a hypoechoic mass larger than 5 mm, or bile duct wall thickness greater than 3 mm with an irregular outer margin 17 .

### Statistical analysis

Predetermined sample size was calculated considering a significance level (α) of 5% (Z _α/2_ = 1.96), an accuracy of 91.7%,(9), and a clinically relevant difference (d) of 0.09 and the following formula 18 :

$$n=\frac{{\left(Z_{\alpha /2}\right)}^{2}\;\times V(\hat{AUC})}{d^{2}}$$

where $V\left(\hat{AUC}\right)$ is variance of estimated AUC and is equal to (0.0099×e ^-a^2/2^ ) × (6a ^2^ +16), a=φ ^-1^ (AUC) ×1.441, and φ ^-1^ denoting the inverse of standard cumulative normal distribution. The value of a =NORM.S.INV(0.917) ×1.414, resulting a $V\left(\hat{AUC}\right)$ = 0.056736789 and n=(1.96 ^2^ ×0.056736789)/(0.09 ^2^ ) = 26.9 rounded to 27. Considering a 10% loss to follow-up, n=30 for each group and a total sample size of 60.

Mann-Whitney test was employed to compare patient age and mass size between groups considering the distribution of raw data per group (Shapiro-Wilks test). Analysis of diagnostic accuracy was conducted on an intention-to-diagnose basis 19 . Diagnostic accuracy was assessed using sensitivity (Se), specificity (Sp), positive predictive value (PPV), negative predictive value (NPV), and overall accuracy, with 95% confidence intervals calculated using the Wald method, which were determined using Excel V3 of the Clinical Utility Index Calculator 20 . In addition, we calculated the efficiency index (EI) and inaccuracy index (InI) to evaluate which test performed better, with high EI and low InI indicating superior diagnostic performance 20 . To test for differences in frequencies, the Chi-squared or Fisher's exact test was utilized based on theoretical values. Performance metrics between diagnostic methods and the enhancement pattern were tested with a Z-test for proportions. All statistical tests used in this study were two-sided, with a significance level (α) of 0.05.

## Results

**Fig. 2:**
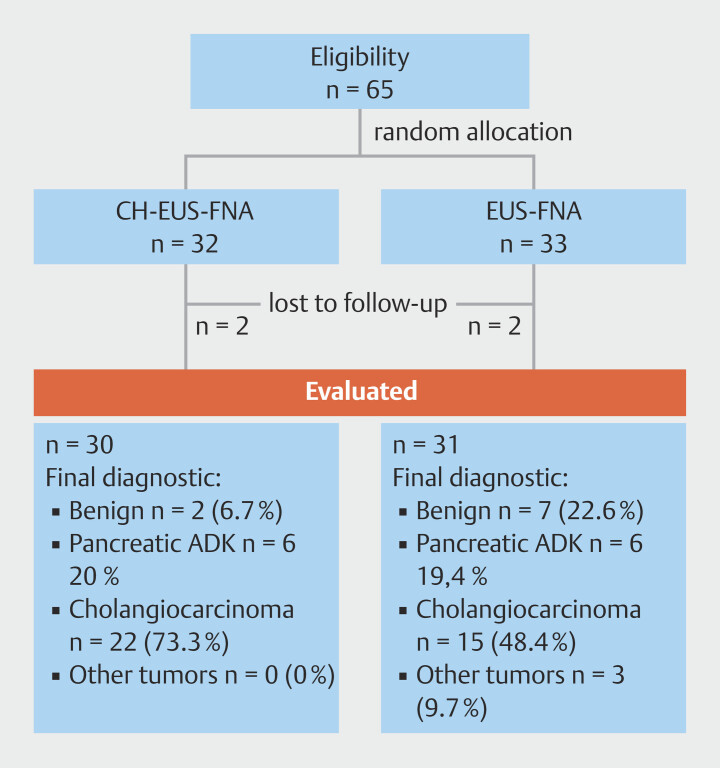
Patient selection flowchart. ADK, adenocarcinoma; CH-EUS-FNA, contrast-enhanced harmonic endoscopic ultrasound-guided fine-needle aspiration; EUS-FNA, endoscopic ultrasound-guided fine needle aspiration.

We evaluated 65 patients with suspected malignant biliary stenosis and 30 patients were included in the CH-EUS-FNA group and 31 patients in the EUS-FNA group ( Fig. 2 ). Patient age ranged from 34 to 89 years, with more men than women, but without significant differences between the groups ( Table 1 ). Mass location, size, and stricture/mass ratio were similar in both groups, although the EUS-FNA group had more biliary plastic stents than the CH-EUS-FNA group. No adverse events occurred following FNA procedures in either group.

**Table:** **Table 1** Demographic and clinical characteristics of evaluated patients.

Characteristics	All n = 61	EUS-FNA n = 31	CH-EUS-FNA n = 30	Statistics *P* value
Age, years ^*^	70 [63 to 75]	70 [65.5 to 73.5]	71 [63 to 75]	0.04 (0.9655)
Men:women ^†^	36:25	20:11	16:14	0.79 (0.3746)
Mass location ^‡^				0.01 (0.9340)
Distal bile duct	43(70.5)	22 (71)	21 (70)	
Proximal bile duct	18 (29.5)	9 (29)	9 (30)	
Mass size, mm ^*^	20 [15 to 25]	20 [11.5 to 24]	20 [18 to 25.8]	-1.43 (0.1532)
Stricture:mass ratio ^§^	39:22	19:12	20:10	0.19 (0.6620)
Stricture:mass ratio by localization ^§^				
Distal	31:12	16:6	15:6	0.01 (0.9244)
Proximal	8: 10	3:6	5:4	n.a. (0.3953)
Biliary plastic stent ^‡^				10.8 (0.001)
No	43 (70.5)	16 (51.6)	27 (90)	
Yes	18 (29.5)	15 (48.4)	3 (10)	
^*^ Data are summarized as median [Q1 to Q3], where Q1 is the 25 ^th^ percentile; Q3 is the 75 ^th^ percentile; comparison between arms with Mann-Whitney test. ^†^ Data are summarized as absolute frequencies; comparison between arms with Chi-Squared test. ^‡^ Data are summarized as absolute (relative %) frequencies, comparison between arms with Chi-Squared test ^§^ Data are summarized as absolute frequencies. *P* value is the probability; n.a. indicated Fisher’s exact test. EUS-FNA, endoscopic ultrasound-guided fine-needle aspiration; CH-EUS-FNA, contrast-enhanced endoscopic ultrasound-guided fine-needle aspiration.				

Final diagnosis was established based on either EUS-FNA or CH-EUS-FNA in 46 patients (75.4%), surgery in eight patients (13.1%), ERCP forceps biopsy in six patients (9.8%), and follow-up in one patient (1.6%). Final diagnoses were cholangiocarcinoma in 37 patients (60.6%), pancreatic ductal adenocarcinoma in 12 patients (19.6%), benign strictures in nine patients (14.7%), neuroendocrine tumor in one patient (1.6%), non-Hodgkin’s lymphoma in one patient (1.6%), and hepatocellular carcinoma in one patient (1.6%).

A higher number of patients in the EUS-FNA group compared with CH-EUS-FNA had a plastic biliary stent, especially with distal localization of tumor ( **Supplementary Table 1** ) but this impeded EUS visualization with removal and reinsertion of stent in only one case.

In the CH-EUS arm, seven patients exhibited hypo/isoenhancement, two with cholangiocarcinoma, four with pancreatic ductal adenocarcinoma, and one with benign strictures. Hyperenhancement was present in 23 patients: 20 with cholangiocarcinoma, two with pancreatic ductal adenocarcinoma, and one benign stricture. The enhancement pattern was not significantly associated with tumor location (Fisher’s exact test *P* = 0.6402) ( **Supplementary Table 1** ).

### Tissue acquisition

False-negative results were present in nine patients: five in the CH-EUS-FNA group (three cholangiocarcinomas and two pancreatic adenocarcinomas) and four in the EUS-FNA group (three cholangiocarcinomas and one pancreatic adenocarcinoma). Diagnostic parameters for CH-EUS-FNA and EUS-FNA were similar, with marginally elevated higher values for EUS-FNA as shown in Table 2 .

**Table:** **Table 2** Comparison of diagnostic accuracy between EUS-FNA and CH-EUS-FNA.

	EUS-FNA	CH-EUS-FNA	*P* value
TP	20	23	
TN	7	2	
FP	0	0	
FN	4	5	
Sensitivity	83.3 (68.4–98.2)	82.1 (68.0–96.3)	0.9014
Specificity	100	100	> 0.999
PPV	100	100	> 0.999
NPV	63.6 (35.2–92.1)	28.6 (0.0–62.0)	0.0061
Accuracy	87.1 (35.2–92.1)	83.3 (70.0–96.7)	0.6758
LR+	DIV0	DIV0	
LR-	0.17 (0.07–0.41)	0.18 (0.08–0.40)	0.9181
CUI+	0.833 (0.692–0.975)	0.821 (0.686–0.957)	
CUI-	0.636 (0.429–0.843)	0.286 (0.000–0.625)	
for case-finding	Excellent	Excellent	
for screening	Fair	Very poor	
EI	45.56	5.00	
InI	0.02	0.20	
CUI, clinical utility index; CH-EUS-FNA, contrast-enhanced endoscopic ultrasound-guided fine-needle aspiration; EI, efficiency index (the highest the value the better the test); EUS-FNA, endoscopic ultrasound-guided fine-needle aspiration; InI, inaccuracy index (the smallest the value the better the test); LR, likelihood ratio; NPV, negative predictive value; PPV, positive predictive value.			

The diagnosis rate obtained through CH-EUS-FNA or EUS-FNA was similar regardless of mass location, enhancement pattern, and stricture or mass-forming type of tumors ( Table 3 ).

**Table:** **Table 3** Diagnostic rate of EUS-FNA and CH-EUS-FNA by tumor location, tumor type (mass-forming vs. stricture-type), and enhancement pattern.

		EUS-FNA (n = 31)	CH-EUS-FNA (n = 30)	*P* value
Location, no./n (%)	Proximal tumors	7/9 (77.7)	9/9 (100)	0.2353
	Distal tumors	20/22 (90.9)	16/21 (76.2)	0.1675
Type of tumor, no./n (%)	Mass-forming tumors	11/12 (91.6)	9/10 (90)	0.7402
	Stricture type tumors	16/19 (84.2)	16/20 (80)	0.8473
Enhancement pattern, no./n (%)	Hypoenhancement	N/A	7/7 (100)	0.1781
	Hyperenhancement	N/A	18/23 (78.2)	
*P* values resulted from Fisher’s exact test, excepting the Enhancement pattern where Z-test for proportions was applied. CH-EUS-FNA, contrast-enhanced endoscopic ultrasound-guided fine-needle aspiration; EUS-FNA, endoscopic ultrasound-guided fine-needle aspiration; N/A, not applicable.				

## Discussion

Regardless of tumor characteristics, this trial demonstrated that CH-EUS-FNA did not improve diagnostic outcomes compared with standard EUS-FNA and it was slightly less efficient for evaluating bile duct tumors.

The European Society of Gastrointestinal Endoscopy (ESGE) recommends tissue acquisition in the case of indeterminate biliary strictures, especially for distal and extrinsic strictures 21 . However, tissue acquisition should be avoided for proximal cholangiocarcinomas (CCAs) in patients who are candidates for liver transplantation (unresectable CCAs less than 3 cm in diameter, with no metastasis or nodal involvement, or CCA in primary sclerosing cholangitis) or curative surgery due to the risk of tumor seeding 22 23 . Preoperative EUS-FNA was found to have no detrimental impact on overall or progression-free survival 24 .

In this study, patients were randomly assigned to two groups: EUS-FNA and CH-EUS-FNA, with similar tumor locations, mass sizes, and stricture-to-mass ratios. Sensitivity was 83.3% for EUS-FNA and 82.1% for CH-EUS-FNA, comparable to previous meta-analyses, which reported sensitivities of 75% for biliary strictures (range 43%-100%) 25 and 73.6% for biliary tumors (range 66.6–91.5%) 26 . Data reported in the specialty literature indicate that presence of a mass on EUS enhances sensitivity of tissue acquisition compared with tumors with a wall-thickening appearance 27 . In addition, EUS is preferred for distal and extraductal lesions, whereas ERCP sampling is favored for proximal and tumors that appear to have wall-thickening 21 . Our results showed that the appearance of either mass/stenosis had a similar diagnostic rate in both groups, although stricture type was more frequently seen than mass-forming tumors. However, the NPV was lower in the CH-EUS-FNA arm compared with the EUS-FNA arm (28.6% vs 63.6%) ( Table 2 ), and a low 47% NPV was reported in a meta-analysis 28 . The difference between the two arms may be related to lesion appearance, because the diagnostic rate for patients with a mass was 91.6% for EUS-FNA versus 90% for CH-EUS-FNA, whereas the diagnostic rate for patients with a stricture was 84.2% for EUS-FNA versus 80% for CH-EUS-FNA.

EUS-FNA demonstrates superior, although non-significant, diagnostic performance compared with CH-EUS-FNA across several critical metrics, with higher value of EI for EUS-FNA than CH-EUS-FNA, indicating more efficient diagnostic capability ( Table 2 ). Furthermore, the InI for EUS-FNA is substantially lower, and the clinical utility index for excluding a diagnosis (CUI-) underscores a marked advantage for EUS-FNA ( Table 2 ), showing that EUS-FNA is equally efficient in clinical practice for tumor differentiation, establishing it as the preferable technique over CH-EUS-FNA. The explanation could be related to hyperenhancement of most of the biliary tumors, which might impede visualization of the needle during actuations.

The diagnostic rate for proximal tumors was lower for EUS-FNA than for CH-EUS-FNA, and higher for EUS-FNA than for CH-EUS-FNA for distal tumors ( Table 3 ), which aligns with data from the literature 29 . The oldest study found a higher sensitivity for distal than for proximal tumors (81% vs. 59%), whereas a more recent one reported the contrary (44% for distal and 91% for proximal) 9 29 . Raine et al. reported on a sample of 97 patients with biliary mass or strictures analyzed retrospectively that the distal location of biliary tumors is associated with an increase of sensitivity to 95% 30 . However, our data proved that both locations are associated with similar results.

This study exclusively utilized 22G needles, aligning with previous findings indicating no significant differences in diagnostic rates among different needle sizes (22G vs. 25G vs. 19G) (70.9% vs. 75.3% vs. 66.7%) and infrequent complication rates 31 . Only EUS-FNA needles were used at the time of patient inclusion, in conformity with 2017 and 2021 ESGE guidelines 32 33 , utilizing two passes per patient, with MOSE assessment ensuring sample adequacy 32 . This represents a limitation of this study, given the data on diagnostic yield in pancreatic masses with EUS-FNB needles 34 . To date, use of EUS-FNB for biliary tumors has been reported in only one study (26 patients with EUS-FNB and 4 with EUS-FNA), with a sensitivity of 73.9% 9 .

Presence of a biliary stent did not diminish diagnostic results in our group, although Raine et al. proved that presence of biliary stents diminished the diagnostic rate (odds ratio = 0.14, *P* = 0.004), by decreasing accuracy of EUS-FNA from 95% to 65% in distal lesions, and from 86% to 56% in perihilar lesions 30 . However, although the number of patients with a biliary stent was higher in the EUS-FNA group than in the CH-EUS-FNA group, the diagnostic rate was similar ( Table 1 ).

The role of contrast in biliary neoplasms has been explored in two trials: one focused on staging 35 and the other on detecting tumor extension 11 . However, no studies have assessed the role of contrast in guiding tissue acquisition in biliary stenosis. Our team previously showed that in pancreatic diseases, there is no advantage for routine contrast guidance in this context 13 .

In CH-EUS, cholangiocarcinoma typically presents with irregular rim-like hyperenhancement at the tumor periphery during the arterial phase, often persisting until the portal phase. In our study, this pattern was observed in 20 of 30 patients analyzed with CH-EUS, all of whom had cholangiocarcinoma. However, two cases were hypoenhanced, indicating that hyperenhancement is not pathognomonic for biliary duct tumors. Considering that features such as ductal and vascular invasion become more discernible after contrast administration, CH-EUS was more accurate than contrast-enhanced CT in detecting invasion beyond the biliary wall (92.1% vs. 45.9%, *P* = 0.0002), but without significant difference in ability to detect invasion of other organs between the two modalities 10 . For T-staging of tumors, CH-EUS demonstrated better accuracy than CE-CT (73.7% vs. 39.5%, *P* = 0.0059) 11 . In addition, EUS tended to have higher accuracy than CE-CT for T-staging, although the difference was not statistically significant (60.5% vs. 39.5%, *P* = 0.052). Due to rapid washout of the contrast agent, CH-EUS was not recommended for N or M staging 11 .

This study has some limitations. First, the number of enrolled patients was relatively small. Nevertheless, the sample size calculation adhered to best practices for diagnostic studies, relying on the expected diagnostic accuracy derived from previous studies and incorporating a 10% margin for potential dropouts. To reduce the possibility of false-negative results, repeated EUS or ERCP were performed in patients with false-negative results. Second, only two passes of FNA were performed, and no EUS-FNB was conducted at time of patient selection, according to hospital policy at the time of patient inclusion. Third, no rapid on-site evaluation (ROSE) was available, but MOSE was utilized to address this limitation, and diagnostic yield was consistent with previously reported data in the literature. Fourth, approximately 20% of included patients had pancreatic adenocarcinoma with common bile duct invasion. This may have introduced bias, because these tumors typically exhibit hypoenhancement, unlike the hyperenhancement characteristic of cholangiocarcinomas. Finally, there were 70.5% distal tumors and a lower number of proximal biliary tumors ( Table 1 ), and we noticed no difference in diagnostic results between different tumor location or features, which is quite different from the contradictory data existing in the literature 9 29 30 .

This study also has several strengths. To the best of our knowledge, it is the study to compare diagnostic performance of EUS-FNA and CH-EUS-FNA for bile duct tumors. We ensured a direct and accurate comparison by excluding patients whose tissue or cytology samples were obtained from sites other than the bile duct. Despite absence of an on-site cytopathologist, sensitivity and accuracy of our EUS-based sampling were comparable to those reported in the literature, even though we exclusively included tissues acquired from the bile duct. This consistency may be attributed to use of advanced EUS imaging technology, high-quality devices, and the expertise of our operators. In addition, we found that EUS-FNA outperformed CH-EUS-FNA in evaluating biliary tumors.

Our study has limitations that must be highlighted. First, the small sample size could be responsible for failure to detect statistically significant differences and may limit the ability to determine the impact of patient-specific characteristics on diagnostic accuracy. Second, use of specific patient selection criteria limits generalizability of the results to broader populations. In addition, the single-center design introduces potential biases that could affect the outcomes. Further large-scale, multicenter studies involving diverse patient populations are needed to validate these findings and to better elucidate the role of CH-EUS in guiding EUS-FNA for staging of biliary stenosis. Despite the listed limitations, our results demonstrated better EI and inaccuracy index for the EUS-FNA method. Our findings suggest that routine use of contrast is not necessary for guiding EUS-FNA in evaluation of bile duct tumors, but this result must be validated in larger multicenter studies that include diverse patient populations to improve generalizability.

## Conclusions

Standard EUS-FNA and CH-EUS-FNA demonstrated comparable diagnostic accuracy in evaluation of extrahepatic bile duct tumors, with similar sensitivity, specificity, and overall accuracy. Although efficiency and inaccuracy indices slightly favored standard EUS-FNA, these differences did not result in a statistically significant improvement in primary diagnostic outcome. Therefore, our findings suggest that routine use of contrast enhancement during EUS-FNA does not offer additional diagnostic benefits and may be unnecessary in clinical practice.

## Supplementary Material

Supplementary Material Zusatzmaterial
