# Supplementary material for: Contrast-enhanced vs. standard endoscopic ultrasound fine-needle aspiration for diagnosing malignant biliary tumors: Randomized controlled trial
Source: Endosc Int Open. 2025 May 12;13:a25698969. doi: 10.1055/a-2569-8969 (PMC12080515; doi:10.1055/a-2569-8969)
Supplement: Supplementary file 1 — Supplementary Material [file 10-1055-a-2569-8969_25704870.pdf]

Supplementary Table 1 Distribution of biliary stents in mass forming/strictures type tumors and in proximal/distal tumors.

	EUS-FNA group		CH-EUS-FNA	
	**Proximal**	**Distal**	**Proximal**	**Distal**
Biliary stents in mass forming tumor (no./n)	2/6	5/6	1/4	1/6
Biliary stents in stricture type tumor (no./n)	2/3	6/16	0/5	1/15
Total	15/31		3/30	
Iso/hypoenhancement (no./n)			3/7	4/7
Hyperenhancement (no./n)			6/23	17/23

Endosc Int Open | DOI 10.1055/a-2569-8969 | © The Author(s).
